# How Best to Establish a Dental Practice

**Published:** 1877-01

**Authors:** E. G. Wheeler

**Affiliations:** Mobile, Ala.


### ARTICLE III.

### *How Best to Establish a Dental Practice.*

BY DR. E. G. WHEELER, MOBILE, ALA.

It is said of the poet that he is "born, not made." To some extent this sentiment is true of the dentist; for there is constantly such a demand for that kind of ability which promptly discovers the relation of cause and effect, and intuitively discerns the shortest and best means to accomplish a given end, that the possessor of such a genius starts out with superior advantages to one who has it not. But since it is also true that he who runs swiftly does not always win the race I would say to every one who has a sound mind in a healthy body, "God speed." The success of many who have had no better capital than this to start upon, is before us for our encouragement; for it generally needs but a judicious arrangement of the materials with which nature has furnished us, duly fashioned by science and art, to construct a specimen of dental architecture praiseworthy to the architect and honorable to the profession.

It gives me peculiar pleasure to review the past and to record the conclusions of a somewhat extended experience, and no little observation, with the hope that it may benefit some one whose professional life is scarce begun; for it is wisely ordered that in the progressive development of man, the experience of individuals may be communicated to others, and as we have profited by the wisdom of the past, so our successors may gather instruction from the records we leave behind.

First, what is success, or the objective point which the aspirant should keep ever in view? for life is insipid without an object, and the more clearly defined it stands out to our earnest gaze, the steadier will be our race.

Avarice defines it *wealth*, and would award the crown to him who has stocks and bonds, houses and lands to exhibit as the reward of his toil. There is such a fascination in display, such an attractiveness in the ease and comfort which wealth sometimes bring, that it is hard to frame an argument to prove it unworthy of our highest ambition; ad yet when it becomes the supreme object, it may be linked with charlatany and dishonesty; qualities which generally defeat the end in view, though sometimes so skillfully played as to deceive those whom they defraud. Surely, then this cannot be success, neither can it be found in any other object concentrated in self. The true dignity of our profession presupposes that we are not altogether selfish, but that we, in common with our medical brethren, labor to relieve the distressed, to palliate, and, if possible, heal the maladies incident to humanity—to search out from the hidden mysteries of nature, laws which govern health, that disease, and consequently pain and suffering, may be prevented. Such objects are incompatible with anything purely selfish, and as such they ennoble and dignify our calling.

The first element of success, then, is found in the energizing power of a high and noble purpose to sustain, and if possible, to elevate a profession which has for its object not selfish aggradisement, but the welfare of suffering humanity. This should be the mainspring to move the whole machinery of life, prompt every action and purify every impulse. In the sacred presence of such a principle, dishonesty stands abashed, and quackery hides her blushing face. It carries with it a moral power which sustains us when disappointment comes. Lay the foundation, then, of our architectural design, broad and deep in a just and proper conception of the scope and dignity of the profession. Upon this basis the superstructure may securely stand, and let it be cemented with *Honesty*.

Not that honesty which is so from policy, for that is little better than galvanized rascality; but that honesty which is the offspring of a deep consciousness of responsibility—a principle that would scorn a mean action in oneself, and would surely expose deceit in others. The operator who is

2

truly honest will not enter the field of action until he is fully instructed and properly equipped, lest those who confide in him suffer from his unskillfulness. He will never betray the confidence of his patients by palming off a miserable counterfeit for the genuine article. It will lead him in examination to be accurate, in operations to be thorough and skilled, and in all his intercourse to give unfaltering allegiance to the dictates of an enlightened conscience. Such a principle discourages opposition, commands respect, and secures that confidence without which there is no success.

The foundation being laid and duly cemented, we are now ready for the superstructure, the materials for which will be found under the head of *Education.*

The average dentist of to-day is certainly an improvement over his brother of twenty or thirty years ago—an improvement more perceptible to one whose professional life spans that interval than to one who has recently germinated under the fostering care of his Alma Mater. Then an apprenticeship of a longer or shorter duration in a dental office was a legitimized entrance into the profession; to-day it but fits him for his college course. Then his curriculum was exceedingly limited; now it is greatly extended, embracing many collateral sciences. Then an educated hand was the desideratum; now an educated mind, which guides the hand, enlarges the comprehension, elevates the whole man, and renders him competent to grapple with the grand physiological and pathological questions which are constantly arising.

Education is the vital question of the day. It bears on its front the weal or the woe of future generations; for, in proportion as the standard is high, will be drawn into our professional ranks men of corresponding talents and capabilities, who will become its exponents, and in proportion as the standard is low will it be the resort of ignorance and stupidity. It is a grave mistake for any one to discourage the idea of a medical education being important as the basis of our specialty. I would go even farther, and say, better still that every candidate for the ranks of our profession could bring with him not only a thorough medical training, but also that discipline of mind which it is the province of our literary colleges to furnish ; then would our institutions not be straitened for gifted teachers, or our literature for instructive contributors. We should then march forward with the proud stateliness of a thoroughly disciplined army to conquest and victory. Then we should have less need of legislature enactments to guard the portals of our profession, for ignorance and incompetency would shrink from a contact which would only reveal their deformity.

Is this ideal picture attainable? Whether it is fully or not, we shall be found marching in that direction, if we but inspire each new candidate for professional honors with the laudable ambition to become a standard-bearer, personating the beautiful conception of our honored poet, as he mounts the Alpiny crags of error and ignorance, bearing aloft his unfurled banner, inscribed, "Excelsior," until it be placed on the eternal rock of truth, and in the full sunlight of an exalted pre-eminence.

The discussion of this question cannot be otherwise than productive of good. Already we can see the crest of the incoming wave of popular opinion, demanding a superior quality of work and a more intelligent treatment. The revolution has commeneed, and happily revolutions never go backwards.

It is not impossible that we who are now upon the stage of action may in the future witness a substantial advancement in our professional status, in respect of education which will astonish us still more than the past.

It has been the writer's pleasure for three years to attend the regular lectures of a medical college, and in response to the oft-repeated inquiry, "cui bono?" the answer would come impromptu from a heart grateful for the opportunity: the *good* of those who favor me with their patronage ; the *good* of the profession which claims my energies and my affections.

Let him who would *best* build up a dental practice heed the maxim, "knowledge is power." A superior knowledge of dentistry as a specialty gives him eminence among dentists; but if, added to such special training, he has the broader culture of the physician, the medical fraternity will show their affinity for him by giving him admission to their counsels, consulting him in cases of oral surgery and by throwing around him generally the ægis of their protection; and who can estimate the power of their patronage and their influence?

What is meant, then by a dental education, is the very largest amount of physical and mental culture possible, sufficiently comprehensive to cover the broad foundations upon which we are building.

Our structure thus far has more of solidity than beauty. It needs ornamentation to give it attractiveness. A finished and classical style of architecture demands columns and cornice of height and strength proportionate to the edifice. These we shall name *Diligence* and *Thoroughness*.

The best instructor in wisdom has said, "The hand of diligent maketh rich," and again, "Seest thou a man diligent in business, he shall not stand before mean men," and observation teaches us that he who would reap the reward must endure the toil. The veteran who has achieved success may occasionally lay his armor down and rest from his toils, not so the youthful warrior whose laurels are yet to be won. A dental practice is built up by a gradual accretion of patients. Each well and thoroughly treated patient brings another, and that other a third, and so on progressively until the office hours are filled. The same rule holds good in retrogression. A neglected patient communicates his disappointment to another, a third listens to the story of inattention, and they all seek elsewhere for one whose systematic diligence can be relied upon. There is a feeling of security in trusting one who is always found at his post. It is a Spartan quality which carries with it the assurance of kindred virtues, which together make up the supporting columns of a reliable and substantial character.

Of equal importance is thoroughness. The sod of the valley oftentimes conceals the errors of the physician; not so with the dentist, for as the entablature of a Grecian temple proclaims from afar the merit or demerit of the architect, so the very conspicuousness of our work makes it noticeable by the most casual observer. A want of thoroughness proclaims its own defects, and sooner or later will react with terrible effect upon its author.

It should be the aim of every practitioner to make each new operation superior to the last, constantly pressing forward towards a higher mark; grasping the idea that mediocrity may be excusable in a beginner, but unpardonable in the experienced. The habit once acquired will not easily be neglected; for like other virtues, it has its own reward in the pleasing satisfaction of increasing excellence, and in the well-merited praise of those who are its beneficiaries. In a profession where there is such an opportunity for æsthetic display, such scope for skillful manipulation, it is the height of venality to have no weighter motive than paltry gain. It is to descend to the rank of a hireling when the honor of the master is within our reach. I would then charge him who builds, that no matter how perfect or abundant the materials, nor how elegant the design, if there is a want of thoroughness in their adjustment the structure will surely crumble to the ground, and remain a monumental pile to the disgrace of the ignoble architect.

Our design approaches completion, but the elegancies of modern taste require the display of frescoes, mouldings and tapestry. These we name *Courtesy*, a Christian grace, by which the angularities of character are toned down into symmetry and beauty, just as barren walls and gloomy recesses acquire gracefulness from artistic decorations.

Courtesy often wins its ways to success, even when there is a woeful deficiency of true merit; but when it supplements a character already meritorious it becomes invincible.

There are many reasons why the dentist should especially cultivate this quality. His practice brings him into intimate relations to the weaker sex, towards whom courtesy is always due, and particulaly so when they place themselves under his care, trusting to his gentlemanly bearing for kind and respectful treatment. Under such circumstances it is inexcusable grossness to offend, by word or act, that delicacy of feeling which belongs to them by intuition; besides, the very nature of the services performed are often a terrible tax on their powers of endurance; it is then of more importance that our conduct should not increase the sufferings which are unavoidable.

Every practitioner is to some extent the guardian of our professional honor, and as such he is bound by all the rules of justice and propriety to maintain inviolate a reputation for courtesy, lest he reflect dishonor upon his associates. Sir Walter Raleigh by a single act of polite gallantry won the notice and the patronage of England's proudest queen. Such opportunities are rare; but each one may, by a studious attention to the essentials of good breeding, secure the favor of those whose patronage is more constant than that of queens—increase his self-respect and honor his profession.

Another quality remains to be mentioned, which is equally necessary in a perfected edifice and a perfected character, viz., *Cleanliness.*

Wesley places this next to Godliness, and surely we should not estimate lightly what is second only to the essence of all good. There may not be any great amount of sin in soiled hands and unkempt hair, but there is an infinite amount of disgrace. Slovenly habits may not be a crime recognized by the civil code, but they have their penalties notwithstanding.

They are found in the disgust of the well-bred, the loss of patronage, and a general degradation of character. There are many fastidious persons who shrink from entering a dental office, and dread to think of, much more to see, the implements of the craft. To calm the agitation of such sensitive natures, cabinets should conceal the instruments from view, and when necessarily exposed they should bear no remainder of former service, but on the contrary, from their polished surfaces, appear beautiful rather than dangerous. We can hardly overestimate the importance of a pleasant office tastefully arranged and neatly kept, in calming the apprehensions of those who are so unfortunate as to need our services. By such surroundings we shun the inquisitorial practice which, in the refinement of its maliciousness, first tortures the mind with horrible sights, and then racks the body. Habits of cleanliness are oftentimes the counterpart of inward purity, and if we would aspire to the fellowship of the virituous and pure, we should at least give this proof of worthiness, by possessing and habitually practicing this most Christian virtue.

Let us pause a moment and view the structure thus far completed. The foundations have been laid in a noble purpose, cemented with honesty—we have reared upon it commodious apartments from material furnished us by education. Before us rises the massive entablature of thoroughness—supported by the stately columns of diligence; within and without it is embellished with all the artistic beauties of courtesy and now swept and garnished it stands before us—but still incomplete. It lacks the lofty dome with its graceful curves to accomplish the design. Upon this we write *Consecration*, and as its pinnacle points heavenward, let it signify that all our talents and all our labors should be dedicated to God. Let him who builds catch the inspiration of worthy aims and pure motives from—he then may rest assured that when the architect shall cease from his labors, his work will remain a beautiful temple to commemorate his successes and his virtues.—*Dental Miscellany.*

